# Supplementary material for: Chromothripsis during telomere crisis is independent of NHEJ, and consistent with a replicative origin
Source: Genome Res. 2019 May;29(5):737–49. doi: 10.1101/gr.240705.118 (PMC6499312; doi:10.1101/gr.240705.118)
Supplement: Supplemental Material [file supp_gr.240705.118_Supplemental_file_1.zip › contigs/annotated_contigs/DB57/contig.2.DB57_length_727_mean_cov_10.1801925722.docx]

**DB57_length_727_mean_cov_10.1801925722**

```
CCCTGCTTGTGAAATAAAAGCCTTTTCATTTAATTTCTGCATGACCTTGTCTAGTCTCTTCTCCTTCATGTACTATCTTAAATTTTCCT
 >chr7:144162753-144163090 + E=6e-191
TTCGCTGAGCACCTGGGAGGTTACCTTTGGTAAAGTTCAAAAGCCGGAAATATCGGAGTGAAGGTTAGGTACTATTAGAAGTCAATTTT

CCATTTATTTGTGCTTGGGAAGATCTAGTCAAATTAACCAGATGACTGAATCCTTGAATTTACAGAAAGCCCTTGAAGTTCTGAAAGAT

TTGCCATGTAAATCCCTCTAAGATTCTGGCTCAATCTTGATCAATTCAGCCAGACCAACTGGTCCACTCA|TA|ATAGGTATTTTCTGA
                                                                         >chr7:14466372
TTCTCACCCTCCTCCCACCCTTCACCCTCAAGTAGGCCTCAGTGTCTATTGTTCTTGTCTTTGTGTTCATGTGTACCTAATGTTTAGTT
5-144664113 - E=1e-218
CCCACTTACAAGAGAGAACACACAACATTTGGTCTTCTGTTCCTGCATTAATTGACTAAGAGTAATGGCTTCCAGCTCCATCCCTGTTG

CTGCAAAGGACATAATCTCATTCTTTTCCACGGCTGCATATATGCCACGTTTCCATGGTGTATACATACCACATTTTCTTTATCCAGTC

CACCACTGATGGATATCTGGGTTGATTCCATGTCTTTGGTGTTGTGAGTAGTGCTGTGATGAACATATACGTGCATGTGAATATATGGT

CCCTTATACCACTCTTA
```
